# Development, feasibility, acceptability and potential effectiveness of a healthy lifestyle programme delivered in churches in urban and rural South Africa

**DOI:** 10.1371/journal.pone.0219787

**Published:** 2019-07-31

**Authors:** Catherine Elizabeth Draper, Simone Annabella Tomaz, Ganzamungu Zihindula, Christopher Bunn, Cindy M. Gray, Kate Hunt, Lisa Kim Micklesfield, Sally Wyke

**Affiliations:** 1 Division of Exercise Science and Sports Medicine, University of Cape Town, Cape Town, South Africa; 2 MRC/Wits Developmental Pathways for Health Research Unit, Faculty of Health Sciences, University of the Witwatersrand, Johannesburg, South Africa; 3 Africa Health Research Institute, KwaZulu-Natal, South Africa; 4 University of KwaZulu-Natal, Durban, South Africa; 5 Institute for Health and Wellbeing, University of Glasgow, Glasgow, United Kingdom; 6 Institute for Social Marketing, Faculty of Health and Sports Sciences, University of Stirling, Stirling, United Kingdom; Teesside University/Qatar Metabolic Institute, UNITED KINGDOM

## Abstract

Rising levels of obesity in South Africa require innovation in community-level lifestyle change programmes. Our aim was to co-develop *Impilo neZenkolo* (‘Health through Faith’), a healthy lifestyle programme for low-income, black South Africans delivered through churches, and evaluate its feasibility, acceptability and potential effectiveness. In the first phase we developed programme materials with church members. In the second phase we trained lay leaders to deliver the programme and assessed feasibility, acceptability (observation, focus groups and interviews) and potential effectiveness (pre and post measurement of weight, hip and waist circumferences, blood pressure, self-reported physical activity, dietary habits, health status, self-esteem, psychological distress). The study was conducted in four churches in urban and rural South Africa. The development workshops led to increased focus on positive benefits of participation, widening inclusion criteria to all adults and greater emphasis on Christian ethos. Challenges to feasibility included: recruitment of churches; scheduling of programme sessions (leading to one church not delivering the programme); attendance at the programme (63% attended more than half of the 12 weekly sessions); and poor programme fidelity (in particular in teaching behaviour change techniques). Aspects of the programme were acceptable, particularly the way in which the programme was aligned with a Christian ethos. There was some indication that amongst the 42/68 (62%) for whom we were obtained pre- and post-programme measurements the programme has potential to support weight loss. We conclude that a healthy lifestyle programme for low-income, black South Africans, delivered through churches, may be viable with extensive re-development of delivery strategies. These include finding external funding for the programme, endorsement from national level denominational organisations and the professionalization of programme leadership, including paid rather than volunteer leaders to ensure sufficient time can be spent in training.

## Introduction

South Africa (SA), like other African countries undergoing rapid demographic, sociocultural and economic transitions, faces an epidemic of obesity and non-communicable disease (NCD). The South Africa Health and Demographic Survey showed that in 2016, 67.6% of women and 31.3% of men were overweight or obese, and 44.6% of adults had hypertension.[1] In 2017, the national prevalence of diabetes was 5.4%.[2] NCDs [3] and obesity [4] present staggering costs to health systems, reduce quality of life [5, 6] and economic productivity.[3, 4, 6] In low- and middle-income countries, the burden of NCDs on households is substantial [7, 8] through direct costs of accessing health care and indirect costs of the inability to work and consequent absenteeism.[9]

Given that public health systems are strained in SA,[9, 10] the SA Government has identified an urgent need for community- and legislative-level approaches to NCD prevention, including opportunities for weight loss and increased physical activity.[11] However, community members hold divergent, sometimes conflicting, views about weight: large body size is both culturally valued and seen as a risk for ill-health.[12, 13] A recent study of young people in SA, suggested community members are unaware of obesity-associated risks and programmes to reduce obesity.[14] Perceived costs of ‘healthy’ eating [12] and poor access to opportunities for physical activity [12, 13] may limit uptake of community-based weight loss programmes.

There is a clear need for weight loss and healthy lifestyle programmes that: reach those most at risk; go beyond traditional approaches to health education to support long term behaviour change; and ensure appropriateness and ongoing engagement of community members by drawing on local sociocultural practices. Few such interventions exist in SA: a community-based intervention to increase physical activity was effective, [15] a group-based diabetes management programme, was ineffective,[16] and a church-based lifestyle programme targeting blood pressure and blood glucose has yet to report outcomes.[17]

Faith-based health care providers can have a role in delivering health care to some of the poorest people in Sub-Saharan Africa,[18] and SA church leaders recognise their potential role in prevention of NCDs.[14] Church-based health promotion interventions have been developed and evaluated, mainly in African American populations in the US, demonstrating both reach into under-served communities and some promising weight loss, dietary and physical activity outcomes.[19–21] However, recent systematic reviews, one of obesity interventions [19] and two of physical activity interventions [20, 21] conclude that a lack of sufficiently high quality research evidence hampers programme development in relation to both intervention content (lack of theory, poorly specified) and evaluation (short-term follow up, lack of rigour in evaluations). All studies included in these systematic reviews were conducted in high income countries, mainly the USA.

We developed *Impilo neZenkolo* (‘Health through Faith’ [InZ] in Zulu and Xhosa), a healthy lifestyle programme for low-income, black South Africans, delivered through churches. Our approach drew on best practice for church-based health promotion interventions [22] in that the development process combined research-based knowledge on effective weight management and programme development with local knowledge of the sociocultural environment. It also tried to combine the best of ‘faith-based’ programmes which include elements of spiritual teaching, with the best of ‘faith-placed’ programmes, which use churches as the place for delivery of health promotion programmes. [19, 22]

Our approach was also based on our experience of developing the Football Fans in Training (FFIT) programmes in the UK[23–25] FFIT is a cost-effective weight management and lifestyle programme for men aged 35–65 years delivered over 12 sessions by trained coaches in professional football clubs.[24] A randomised controlled trial demonstrated the mean between-group difference at 12 months in weight loss adjusted for baseline weight and club was 4.94 kg (95% CI 3.95 kg to 5.94 kg) and that the programme was cost-effective. Longer-term follow-up to 3.5 years post baseline for the intervention group showed sustained mean weight loss of 2.90 kg (95% CI 1.78 kg to 4.02 kg) and the programme remained cost-effective.

The FFIT programme was developed to tackle the problem men’s reluctance to take part in traditional weight management programmes.[23] Our research has demonstrated that the culturally-valued context of the professional football clubs holds social and symbolic meaning for participants and serves as the initial ‘hook’ to attract men at high risk of ill health who would otherwise not engage in weight management.[26, 27] It harnesses their desire to ‘do something’ for their health and draws them in with the chance for an ‘insider view’ of the club. Men then remain engaged because of their enjoyment of being with ‘people like me’, and a team spirit generated through interactive adult-learning delivery style, in a valued, masculinised, context.[26, 27] As others argue,[28, 29] the interaction between intervention content (the practice of evidence-based behaviour change techniques and simple information), and the context of delivery (the football club, which attracts men in the first place) are critical to FFIT’s success.[25] It has been successfully adapted for delivery to women [30] and in other professional sports settings with minor changes [31, 32], and has been more substantially adapted in the football setting to emphasise different health outcomes (physical activity and sedentary behaviour).[33]

In developing the InZ programme we tried to apply the generalizable lessons learnt from research on the FFIT programme into a very different setting. We attempted to reach those most at risk by basing the programme in low income settings with the initial ‘hook’ being delivery in church settings (that is, as in the USA, [19, 22] we hoped the church-based setting would attract those who would not otherwise consider weight management). This made sense, because in SA, 84% identify as Christians; amongst black South Africans, 59% attend worship services at least weekly, and another 21% attend once or twice per month.[34] As in FFIT, we wanted to go beyond traditional approaches to health education by attempting to teach the use of evidenced-based behaviour change techniques, and by including information presented simply using adult learning methods (basing teaching on existing knowledge and encouraging mutual vicarious learning). Finally, we attempted to ensure ongoing engagement and appropriateness to setting in a faith-based rather than faith-placed intervention by weaving Christian values, prayer and faith-based singing and mutual support into weekly group sessions.

The overall aim of the research was to consider whether the ‘hook’ of the church, as a culturally valued setting, is a good site for delivering a group-based lifestyle change programme. Our objective, in this paper, is to report on a two phase study, the development of *Impilo neZenkolo* through development workshops with church leaders and members (Phase 1) and its initial evaluation in relation to feasibility, acceptability and potential effectiveness (Phase 2).

## Materials and methods

### Study design and settings

The study was conducted in low-income settings in SA, one urban (a ‘township’) and one rural. Both settings are characterised by low levels of education, high unemployment, and poor access to services, and high prevalence of overweight and obesity, amongst women in particular. In addition, the rural setting is particularly deprived and has high levels of HIV, tuberculosis and NCDs.

Ethical approval was obtained from the University of Cape Town Human Research Ethics Committee (199/2016) and the University of KwaZulu-Natal Biomedical Research Ethics Committee (BFC394/16). Prior to data collection an information sheet was read to participants and written consent obtained. An exception to this was for observation of programme sessions. At the first observation, an information sheet was read to participants and their permission to observe the session verbally agreed. The information made it clear that participant could refuse the observation, in which case the observer would exclude anything they said or did from fieldnotes.

### Phase 1—Recruitment of churches, development of programme materials, and recruitment of programme leaders

#### Recruitment of churches

We aimed to recruit four churches, two in each setting. Inclusion criteria were: church in existence for more than years (to avoid instability in the congregation); churches having >300 people regularly attending (to facilitate adequate recruitment); and church leader(s) being committed to collaborating in programme development and evaluation without payment.

In the urban setting, a research team member (CD) used local knowledge to identify two potential churches to take part. Each was known to have been in existence for more than five years and to have large (>300) congregations. CD is a member at one of these churches and the second was known to be supportive of health and wellbeing in its teaching. CD made initial contact with the leadership in each church and asked them for initial permission to work in the church and to identify potential InZ programme leaders. Having identified potential leaders in each church, CD then made numerous email and telephone contacts to arrange an initial meeting with those potential programme leaders about the study, and to secure agreement to participate in the research.

In the rural setting, through the Africa Health Research Institute’s community engagement team, SW convened a meeting with leaders of 33 Christian churches to assess initial interest in the programme and research. Leaders of 11/33 churches reported an interest and were telephoned to assess whether their church met inclusion criteria. Of four churches meeting the criteria, two were selected based on the enthusiasm of church leaders and commitment to participate fully in programme development workshops.

#### Development of *Impilo neZenkolo* materials

We developed *Impilo neZenkolo* materials in two development workshops in each church (July and December 2016). Participants in these workshops were church leaders and members invited by the church leaders based on who they thought had the enthusiasm to champion the programme and had potential to gain experience from it. In the urban area, workshops were conducted in English and, for ease of scheduling, were held with participants from each church separately; in the rural area workshops were with participants from both churches together and conducted in a mixture of Zulu and English. They lasted between 2–4 hours. We held a subsequent, separate, workshop with development group members in the urban setting, to remind them of the proposed programme and to plan training in January 2017. This meeting was not needed in the rural area, where church members were keen to begin training.

As there were no existing weight management programmes of known effectiveness for this population in SA we had to develop a new programme. We based initial *Impilo neZenkolo* materials on core components of FFIT [23] and on information from the revised food-based dietary guidelines for SA.[35] We chose FFIT as the starting point for InZ, rather than one of the church-based health promotion interventions from the USA, for three main reasons. First, FFIT is very effective and cost-effective and the core components have been clearly identified.[23–27] Second, although Church-based health promotion programmes designed for African Americans are promising [19–21] none are as effective in achieving long term behavioural change as the FFIT programme and programme content is poorly specified.[19, 21] The final reason is very pragmatic. Our team had developed FFIT and our research funding allowed us to explore how it needed to be adapted to the SA church setting.

The development workshops aimed to provide participants with experience of core components of InZ sufficient so they could suggest adaptations to better suit the sociocultural environment and support the ethos and spiritual teaching of the church. The original four core components designed to be delivered in ‘classroom’ sessions were: information presented simply and learnt through peer interaction in an adult-learning style; becoming skilled in behaviour change techniques; a mutually supportive atmosphere and ongoing social support; and using familiar, culturally appropriate, Christian practices such as prayer and singing to promote ongoing engagement. A fifth original core component was a progressive physical activity programme based on a walking programme and group-based exercises.

Workshops included experience of an ice-breaker exercise (sharing favourite Bible verses), information to promote understanding the health benefits of losing weight; a practical application of the UK’s Eatwell healthy eating plate (using plastic/actual portions of food appropriate to the South African setting), [36] self-monitoring (using a pedometer for walking and weighing scales for weight), goal setting and action planning. We had developed the physical activity programme for use in the InZ sessions and at home and included in the workshops sessions on leading physical activity sessions safely. We also sought views on: the best ‘hook’ for potential *Impilo neZenkolo* participants; recruitment strategies; inclusion criteria; whether groups should be single sex; and how the programme might continue to reflect a Christian ethos.

Workshop notes were taken by CD, ST and SW and summarised to inform a week-long programme development meeting during which the full team finalised the *Impilo neZenkolo* materials, including *Impilo neZenkolo’s* theory of change.

#### Recruitment of programme leaders

Church leaders were asked to identify which development workshop participants would like to be programme delivery leaders. They were asked to emphasise that leaders would not be paid, but would learn valuable skills.

### Phase 2 –Assessment of feasibility, acceptability and potential effectiveness of the *Impilo neZenkolo* programme

#### Feasibility and acceptability

We used the number and characteristics of recruited participants to assess the recruitment procedures. To assess acceptability and the extent to which *Impilo neZenkolo* could be delivered as intended, we observed as many delivery sessions as possible with the resources available. Observers (CD, ST. MD and GZ) wrote descriptions of how core components of each weekly session were delivered, which leader(s) delivered it, how members responded, and estimated the time taken for the activities. Attendance sheets were completed for each session.

To further assess acceptability and gain insight into feasibility, we conducted focus group discussions with programme members on completion of the intervention. Topics included motivations for joining, perceived impacts on their lives/behaviours, experiences of doing the programme and views about potential improvements. We also conducted interviews or group discussions with programme leaders post-programme. Topics included recruitment and training as leaders, experience of delivering the programme, and perceptions of: the programme and its outcomes; barriers and facilitators to delivery in their church; and the programme’s sustainability. Finally, we interviewed church leaders. Topics included recruitment, perceptions of the programme and of barriers and facilitators to delivery in their church, and suggestions regarding future implementation. Focus groups/interviews were facilitated by CD and ST in the urban setting and GZ and a trained fieldworker (MD) in the rural setting.

We also asked members to complete a post-programme questionnaire to assess the acceptability of key aspects of the programme:

- Usefulness of 19 programme elements they should have experienced during sessions (1–5 scale from ‘not useful at all’ to ‘very useful’);
- Reasons for missing sessions;
- Rating of the programme and programme leaders (on a scale of 1–10); and
- Likelihood of remaining active after Impilo neZenkolo (1–4 scale from ‘not likely at all’ to ‘very likely’).

#### Potential effectiveness

We assessed potential effectiveness prior to and following programme participation. Objective measurements were undertaken by trained fieldworkers. With participants wearing light clothing and no shoes, fieldworkers measured height, recorded to the nearest mm using a portable stadiometer and weight, recorded to the nearest 100g using digital scales. Height and weight were used to calculate body mass index (BMI). Hip and waist circumferences were each measured three times and the mean calculated. The procedure for both measurements was to ask participants to stand upright, in light or tight clothing, with their feet and heels together. Fieldworkers then squatted beside participants. For hip measurement fieldworkers placed the tape around the most protruding part of the buttocks whilst for waist measurement they placed the tape horizontally around the participant between the iliac crest in the mid-axillary plane and the lowest rib margin and measurement taken at the end of normal expiration.

Blood pressure (BP) was measured following a 5-minute period of sitting, using an Omron BP monitor (model HEM-907). All participants were measured three times with two minutes between each measurement. If the first reading was more than five mmHg more than the second and third then a fourth measurement was taken and the first disregarded. For analysis, we calculated the mean of three valid measurements. Everyone with an elevated BP reading was advised to have their BP checked in a clinic, and given a letter to take with them.

During measurement sessions trained fieldworkers administered a questionnaire which included:

- Sociodemographic characteristics (education level, employment status, household assets) (pre-programme only);
- Church attendance (frequency per week; pre-programme only);[34]
- Self-reported physical activity and sitting time (Global Physical Activity Questionnaire);[37]
- Food habits (including frequency and volumes of food consumption);[38]
- Self-esteem (Rosenberg Scale);[39]
- Health-related quality of life (five-level EuroQoL questionnaire (EQ-5D-5L); [40]
- Psychological distress (Kessler Psychological Distress Scale).[41]

Data were collected in the language with which participants were most comfortable. Programme leaders (hereafter ‘leaders’) from the churches and participating congregation members (hereafter ‘members’) are not differentiated for the outcome measures and are collectively referred to as ‘participants’ in the paper, since leaders not only led but also participated in the programme. Hence leaders’ outcome measures are included with members’ outcomes.

### Data management and analysis

#### Qualitative data

Focus groups/ interviews were transcribed, translated into English, where necessary, and analysed using a thematic framework approach.[42] The main themes and sub-themes were:

- Feasibility: recruitment, training and delivery of key aspects of the programme and member response to programme materials;
- Acceptability: leaders’ and members’ perceptions, including alignment of health and faith;
- Potential effectiveness (leader/member perceptions);
- Recommendations for programme adaption.

The coding framework was applied to the transcripts using NVivo 11 for Mac (QSR International, Doncaster, Australia) by CD and CB. Relevant data extracts for each sub-theme were identified and collated, and summaries were generated for each sub-theme to provide an interpretation of the data.

Field notes for each week observed were summarised by SW and CB in relation to whether the key points for delivery and weekly plan were delivered with fidelity as intended (three categories: not delivered, delivered somewhat, and delivered well). We used excel to manage data and coded specifically delivery of each of the four core components (information content and style, teaching behaviour change techniques, support of a mutually supportive atmosphere, and using Christian practices of prayer and singing). We also noted whether and how well physical activity sessions were delivered and whether the Healthy Lifestyle messages were encouraged (see description of programme below). Learning on feasibility, acceptability and perceived effectiveness from all qualitative data sources was combined in a table under the headings: self-monitoring, goal setting, social support, information and interaction for mutual learning, and fidelity. Illustrative extracts are provided in the text of the paper. Further examples are provided in S1 File, Further qualitative data extracts.

#### Quantitative data

Data were collected and managed using REDCap electronic data capture tools hosted at the Africa Health Research Institute.[43] GPAQ data were cleaned following the WHO STEPS surveillance manual.[44] A wheelchair-bound participant’s GPAQ data were excluded. Questions on food habits included: how regularly the participant ate breakfast during the week; and frequency of consumption of certain foods, including high fat items (chicken/poultry with skin, high fat red meat, butter and margarine) 14 specific fruits and vegetables. A fruit and vegetable score was calculated by summing values recorded on a scale of 0 (never) to 5 (three times daily).

Statistical analyses were undertaken using Stata13 (STATA Corp, College Station, TX) for Mac. Differences between baseline and post-programme measurements for the total sample were assessed using paired t-tests (normally distributed data), Wilcoxon signed-rank analyses (not-normally distributed data) and Pearson’s chi-squared tests for categorical data. GPAQ data were not compared statistically pre- and post-programme and are presented for descriptive purposes only because a large number of participants did not have valid GPAQ data following data cleaning (of 43 participants with GPAQ data post-programme, only 27 were valid according to the WHO STEPS surveillance manual).[44]

## Results

### Phase 1—Recruitment of churches, adaption of programme materials and recruitment of programme leaders

#### Characteristics of churches and participation in development workshops

All recruited churches (n = 4) were protestant (n = 3 Pentecostal) (Table 1). All offered weekly activities as well as Sunday services, including women’s bible reading, men’s prayer meetings, and children’s Sunday school services.

**Table 1:** Characteristics of the participating churches.

Church No	Setting	Denomination	Other features	Participation
**1**	**Urban**	Protestant—part of large global network	Church’s approach has an emphasis on diet and health	Phase 1
**2**	**Urban**	Protestant–Pentecostal, part of wide global network	Church’s approach is charismatic with emphasis on social justice	Phase 1 and 2
**3**	**Rural**	Protestant–Pentecostal, independently run	Church’s approach is charismatic, with emphasis on whole community participation and supporting most needy	Phase 1 and 2
**4**	**Rural/township**	Protestant–Pentecostal, originally part of the largest Pentecostal denomination in SA, which decentralises control to local churches	Church split from main denomination approximately half way through the programme, establishing an independent church with ~20% of the congregation	Phase 1 and 2

Leaders and/or members of all four churches participated effectively in development workshops with approximately seven participants from each rural church and five from each urban church. Although Church 1 members were supportive of the programme, and potential leaders were trained to deliver it, it proved impossible to find a suitable delivery time (discussed further below). Church 1 withdrew from the study and did not contribute to Phase 2. We conducted a follow-up interview with Church 1 leaders to understand these difficulties further.

#### Workshop development of programme

Development workshop participants were generally positive about the core components of the programme experienced and suggested useful minor modifications. In both settings, they suggested greater focus on the health benefits of eating well and being physically active (less stress, improved wellbeing) in addition to the links between obesity and NCDs.

In relation to behaviour change techniques, participants in both settings thought self-monitoring of steps (using pedometers) and weight (using scales) would work well. It was thought that members might not want to share their weight measurements with others, but were likely to use electronic scales if placed discreetly. Participants understood SMART goal setting and considered this easy to teach in the urban but not the rural setting; here participants thought very practical examples and practice of good SMART goals would be needed.

In relation to understanding food and healthy eating, participants in the urban (but not rural) setting thought people in their churches were already quite well informed about food groups. Participants in both settings thought information about portion sizes would be novel and important. They praised the very tangible way that food types and portions were introduced (using plastic/actual portions of food) in the group activity around the Eatwell plate but highlighted the importance of using commonly eaten foods, such as greens and pumpkin. Participants (particularly from the rural churches) enjoyed and valued an activity designed to increase skills in reading food labels.

Participants in both settings agreed it would be relatively easy to teach the physical activity exercises safely, if good information and pictures of “‘dos’ and ‘don’ts” were included in programme materials. They suggested it might be better to deliver exercises in the form of dancing and singing–drawing on practices that feature strongly in their church cultures.

Participants thought the best ‘hook’ to recruit and engage programme participants was an emphasis on Christian ethos and living well as ‘God’s way’. They agreed that encouragement from the Pastor would facilitate attendance. Participants conveyed enjoyment of, and support for, programme components that drew on Christianity. There was a firm conviction, in both settings, that the programme should be open to all adults who wanted to make changes to their lifestyle and not only overweight people, and there was no great demand for single sex groups. Messages on healthy lifestyles were thought relevant for families and the whole congregation, not only programme participants. In the rural area, where fewer speak English, participants were clear that programme materials should be translated into IsiZulu. A suggestion that members in the urban setting would value the programme more if asked to pay even a nominal sum for participation was firmly rejected in the rural setting, where there was a conviction that members should not be asked to pay to ‘be healthy’. Finally, in the urban setting in particular, participants said leaders’ training sessions would need to be short to accommodate their busy schedules.

#### Programme description

Drawing on insights from the development workshops, we decided to: promote *Impilo neZenkolo* as a group-based, free of charge, healthy lifestyle programme with a strong Christian ethos, open to all; and recruit from church congregations, promoted by pastors/church leaders. The final programme comprised 12 sessions, designed to be delivered over 60–75 minutes, once per week, using an adult-learning approach based on what is already known and emphasising social support. Materials included a programme leader manual and programme member manual which gave detailed lesson plans to leaders on what and how to deliver each weekly session. The programme member manual was translated into IsiZulu in the rural setting.

Each session had an educational (‘classroom’) component covering diet, physical activity and weight loss, and behaviour change techniques; and a practical group-based physical activity component, where members were encouraged to go at their own pace and avoid over-exertion. Physical activity materials included an incremental walking programme (focussing on both step counts and intensity of walking), and warm-up, strength and flexibility exercises (illustrated in the manuals). The walking programme was intended to be followed between sessions; suggestions for home-based exercise routines were also offered. The Rate of Perceived Exertion scale was included to try to avoid over-exertion.

Following input from the development workshops, behaviour change techniques were retained, including goal-setting and self-monitoring (daily step counts, weekly self-weighing). Participants were encouraged to make changes to their physical activity and diet that suited their daily routines and preferences, using a ‘personal progress record’ to monitor their own progress. Programme materials emphasised the ongoing practice and review of goals, encouraging practical examples of behavioural goals that could work. They also emphasised: learning to overcome setback, social support and mutual learning through group interaction, and positive feedback from programme leaders and members. Workshop participants had felt that messages on healthy lifestyles were relevant for families and the whole congregation and so we included weekly ‘Impilo neZenkolo Healthy Lifestyle‘ messages for each week that leaders would suggest members share with family members and friends. The messages became a sixth core component of the programme.

Given feedback from the development workshops, every opportunity was used to incorporate a Christian Ethos (e.g. in ice-breakers, singing and prayers at the beginning and end of sessions). Each week key messages, supported by Bible verses, were highlighted to share with family members and the wider congregation. We also referred to participants as ‘members’ (reflecting membership of both Church and programme) and retained the more culturally relevant term ‘leaders’ rather than ‘trainers’ for the programme leaders.

Table 2 details the content of the programme for each of 12 weekly sessions. Table 3 shows the logic model supporting the theory of change for *Impilo neZenkolo*. Programme delivery manuals are available on request.

**Table 2:** Weekly content for the Impilo neZenkolo programme.

	Classroom	Physical activity	Impilo neZenkolo Healthy Lifestyle Message	Bible verse of the week
**week 1**	• Programme overview and introduction to healthy lifestyle • Getting to know one another • Perceptions of control over eating • Importance of regular monitoring of weight • InZ T-shirts	• Learning to walk at a moderate intensity • Introduction to the Rate of Perceived Exertion Scale		*“For where two or three gather in my name*, *there am I with them”*. *Matthew 18*:*20*
**week 2**	• Healthier eating and portion sizes • Comparing personal food diary with a healthy diet • Introduction to the pedometer and self-monitoring of steps • Key message: reduce portion sizes	• Baseline fitness: 6-minute walk test	Reduce portion size of unhealthy foods	“*So whether you eat or drink or whatever you do*, *do it all for the glory of God*”. 1 Corinthians 10:31
**week 3**	• Health benefits of losing weight and 5–10% weight loss targets • Setting SMART goals for healthy eating and walking (steps or intensity) • Importance of physical activity for weight management • Key message: losing a small amount of weight can have real health benefits	• Practising walking at moderate intensity	Maintaining a healthy weight–not too big, not too small–is important. If you are overweight, losing just a small amount of weight–about 5–10% of your body weight, can bring real health benefits.	“*See*, *I am doing a new thing*! *Now it springs up; do you not perceive it*? *I am making a way in the wilderness and streams in the wasteland*.” Isaiah 43:19
**week 4**	• SMART goal review • Importance of physical activity and sitting less for health and wellbeing (and national recommendations) • Overcoming barriers to being active/sitting less • Key message: do 30–45 minutes of moderate intensity physical activity each day	• Introduction to warm up exercises • Practising walking at moderate intensity	It is important for your health to do at least 30 minutes of moderate intensity physical activity on most days of the week. Moderate intensity = slightly out of breath, but still able to talk	“*Do you not know that you are God's temple and that God's Spirit dwells in you*?*”* 1 Corinthians 3:16
**week 5**	• Understanding food labels • Role of sugary drinks in weight gain • Key message: cut down on sugary drinks	• Warm up • Introduction to strength training (upper body)	Cut down on sugary drinks (Drink water instead of fizzy drinks or take less sugar in hot drinks)	“*No temptation has overtaken you that is not common to man*. *God is faithful*, *and he will not let you be tempted beyond your ability*, *but with the temptation he will also provide the way of escape*, *that you may be able to endure it*.” 1 Corinthians 10:13
**week 6**	• Introducing setbacks • Mid-point measures • Shared experiences with InZ ambassador • Key message: setbacks ae normal and can be overcome	• Mid-point fitness: 6-minute walk test • Introduction to strength training (lower body)	Life is full of challenges that can stop us from eating well and being active. These challenges will happen from time to time. Don’t let them defeat you. Just keep going and try to meet your goals.	“*God met me more than halfway*, *he freed me from my anxious fears*.” Psalm 34:4
**week 7**	• Physical representation of group weight loss and fitness • Cooking healthier meals • Key message: use less oil during cooking	• Warm-up • Introduction to flexibility exercises	Use less oil during cooking to improve your health and your family’s health	*“For we are God’s masterpiece*. *He has created us anew in Christ Jesus*, *so we can do the good things he planned for us long ago*.*”* Ephesians 2:10
**week 8**	• Positive and negative social influences on lifestyle • Identifying key supporters • Key message: spread the InZ philosophy to others	• Warm up • Upper body strength exercises Stretches	Everybody needs encouragement and support–spread the healthy eating message and congratulate people who do well	*“Pay careful attention to yourselves and to all the flock*, *in which the Holy Spirit has made you overseers*, *to care for the church of God*, *which he obtained with his own blood*.*”* Acts 20:28
**week 9**	• Popular myths around healthy living • Hints for healthier eating–including fast foods • Key message: eat regular meals and breakfast	• Warm up • Lower body strength exercises • Stretches	Eat meals regularly throughout the day (and always have breakfast if you can)	*“And you will know the truth*, *and the truth will set you free*.*”* *John 8*:*32*
**week 10**	• High risk situations and if-then plans • Key message: make plans to deal with high risk situations	• Home exercise programme • Flexibility exercises • Stretches	Imagine your obstacles to healthy eating and make a plan to overcome them	*“But as for you*, *be strong and do not give up*, *for your work will be rewarded*.*”* 2 Chronicles 15:7
**week 11**	• Reflection on personal achievements (food diary, step count, weight) • End of programme measures • Overcoming setbacks • Key message: congratulate yourself and others when things have gone well	• End fitness: 6-minute walk test • Warm up • Whole body strength exercises • Stretches	Congratulate yourself and others when things have been done well	*“In all your ways acknowledge him*, *and he will make straight your paths*.*”* Proverbs 3:6
**week 12**	• Celebrating achievements: physical representation of group waist reduction & fitness; graduation & end of programme team photo with InZ ambassador • Personal benefits of change • Ongoing SMART goals • Top tips for maintaining change	• Group choice of physical activity	Repeat of all 10 messages	*“Beloved*, *I pray that all may go well with you and that you may be in good health*, *as it goes well with your soul*.*”* 3 John 1:2

**Table 3:** Impilo neZenkolo (InZ) Logic Model.

Inputs	Activities			Outputs	
	Reach	Initiate change	Sustain change	Short term	Longer term
• Relational: - Church leaders and lay programme leaders’ commitment to engage with training and preparation for programme (and sessions) give time free of charge and voluntarily - Church InZ ambassador - Pastors and church elders to drive recruitment • Physical: - materials (e.g. manuals, pedometers, InZ T-shirts, Eat well plate, measurement equipment) • Financial: - funding for materials	People attracted to programme by: - desire to make changes (weight, diet, physical activity) - Church membership and programme’s Christian ethos - pastor’s or other leaders’ ‘advertising’ in church and personal invitation	• Participants welcomed and their commitment valued • Healthy lifestyle and weight loss encouraged in ways that support participants’ identities as church goers (e.g. church-based symbols, activities) and emphasis on positive gains • Culturally-appropriate information • Participants taught and practice behaviour change techniques: self-monitoring weight/stepsgoal setting, review & action planning (diet and PA) • Programme delivered using adult learning styles that emphasise what is already known • Social support promoted: within sessions (e.g. interactive, non-didactic approach) between sessions (e.g. WhatsApp, text, meeting up)	• Skills and competence built through: - encouragement to practice behaviour change techniques - positive feedback • Socially supportive culture promoted among: - participants - wider social networks (church, family, friends, work) • Congregational & community norms influenced through - participants spreading key healthy lifestyle messages - visibility of participants’ healthier lifestyle practices • Understanding of overcoming setbacks and plans for high risk situations promoted • Participants encouraged to recognise personal benefits of changes made	• Clinical: - weight loss, waist reduction, improved blood pressure • Behavioural: - participants’ increased physically activity (amount or intensity) and healthier diets - positive lifestyle changes within participants’ social networks (e.g. other church members, family, friends, work colleagues) • Psychological: - improved self-esteem, wellbeing, health-related quality of life • Sociocultural: - participants feel socially supported by/supportive of lay leaders, other participants and wider church, family and friends’ networks	• Clinical: - participants maintain at least 5% weight loss and improvements in waist circumference and blood pressure - participants show improvement in health outcomes (e.g. type 2 diabetes, cardiovascular disease) • Behavioural: - changes in physical activity/sitting/diet become embedded in participants’ daily lives • Psychological: improved self-esteem, wellbeing, health-related quality of life • Sociocultural: - new congregational & community norms around weight and healthy lifestyle choices

#### Recruitment and training of programme leaders

Thirteen people who participated in the development workshops volunteered to be programme leaders (Church 1 n = 2, Church 2 n = 3, Church 3 n = 4, Church 4 n = 4).

Given feedback in development workshops and on-going problems in scheduling in the urban area, we reduced our intended two-day training to two, two-day-hour training sessions and developed a detailed ‘programme leader training’ manual. This highlighted learning and core skills for delivery and the importance of advance preparation for each session. In the urban setting, the first training session was delivered by ST with leaders from both churches at one church’s premises; the second session was delivered separately in each church’s own premises. In the rural setting, both training sessions were conducted by GZ with leaders from both churches at the Africa Health Research Institute. An additional training session focussing on behaviour change techniques was conducted between sessions 3 and 4 with the four programme leaders from Church 3 after observations of sessions documented that these techniques were not being delivered well in this church. Training materials are available on request.

### Phase 2 –Assessment of feasibility, acceptability and potential effectiveness

#### Response to data collection

We observed, and took detailed notes for, 27/36 possible *Impilo neZenkolo* sessions at the three churches which delivered the programme. Availability of isiZulu-speaking field staff and transport difficulties made observation of all sessions difficult in the rural area.

We conducted: a post-programme focus group discussions with programme members in each church delivering the programme; two focus group discussions (Churches 1 and 2) and six individual interviews (Churches 3 and 4) with programme leaders; and three interviews with Church leaders (usually Pastors) in each church (Table 4).

**Table 4:** Numbers of focus group and interview participants.

Data collection method	Participants	n
**Focus groups**		
Church 1	Programme leaders	2
Church 2	Programme leaders	3
	Programme members	8
Church 3	Programme members	7
Church 4	Programme members	9
**Individual interview**		
Church 2	Church leader	1
Church 3	Church leader	1
	Programme leaders	2
	Programme member	1
Church 4	Church leader	1
	Programme leaders	4
	Programme members	1

Baseline measurements were completed on 84 participants (including 16 participants in Church 1, at which delivery of the programme proved unfeasible because participants could not schedule a mutually convenient time for programme delivery) (Table 5), and post-programme measurements on 42 participants from Churches 2–4.

**Table 5:** Participants’ socio-demographic characteristics (total sample at baseline)*.

	Total (n = 84)	Church 1 (n = 16)	Church 2 (n = 24)	Church 3 (n = 24)	Church 4 (n = 20)
(n, % women)	61 (72.6)	13 (81.3)	15 (62.5)	23 (95.8)	10 (50.0)
**Age**	*n = 80*	*n = 14*	*n = 16*	*n = 22*	*n = 20*
(years)	43.6 (35.6, 50.0)	31.2 (25.6, 36.8)	42.6 (36.2, 45.8)	50.9 (40.2, 53.9)	44.8 (38.0, 48.5)
**Marital status**	*n = 79*	*n = 14*	*n = 22*	*n = 23*	*n = 20*
Single	42 (53.2)	11 (78.6)	16 (72.7)	7 (30.4)	8 (40.0)
Married	23 (29.1)	0	2 (9.1)	13 (56.5)	8 (40.0)
Co-habiting	6 (7.6)	0	2 (9.1)	2 (8.7)	2 (10.0)
Separated	1 (1.3)	0	0	0	1 (5.0)
Widowed	7 (8.9)	3 (21.4)	2 (9.1)	1 (4.4)	1 (5.0)
**Education**	*n = 78*	*n = 14*	*n = 21*	*n = 23*	*n = 20*
Never attended school	2 (2.6)	0	0	2 (8.7)	0
≤ Grade 7	12 (15.4)	0	1 (4.8)	11 (47.8)	0
Grade 8–11	26 (33.3)	6 (42.9)	13 (61.9)	4 (17.4)	3 (15.0)
Completed school	38 (48.7)	8 (57.2)	7 (33.3)	6 (26.1)	17 (85.0)
**Tertiary education**	*n = 73*	*n = 14*	*n = 16*	*n = 23*	*n = 20*
None	38 (52.1)	3 (21.4)	9 (56.3)	20 (87.0)	6 (30.0)
Certificate	19 (26.0)	7 (50.0)	6 (37.5)	3 (13.0)	3 (15.0)
Diploma	7 (9.6)	3 (21.4)	0	0	4 (20.0)
Degree	8 (11.0)	1 (7.1)	0	0	7 (35.0)
Prefer to not say	1 (1.4)	0	1 (6.3)	0	0
**Employment status**	*n = 80*	*n = 14*	*n = 23*	*n = 23*	*n = 20*
Unemployed	50 (62.5)	6 (42.9)	14 (60.9)	21 (91.3)	9 (45.0)
Employed part-time	4 (5.0)	1 (7.1)	1 (4.4)	1 (4.4)	1 (5.0)
Employed full-time	25 (31.3)	7 (50.0)	8 (34.8)	1 (4.4)	9 (45.0)
Prefer to not say	1 (1.3)	0	0	0	1 (5.0)
**Socioeconomic status**	*n = 80*	*n = 14*	*n = 23*	*n = 23*	*n = 20*
Asset score (out of 22)	9.8 ± 3.5	11.1 ± 2.8	9.3 ± 3.4	7.2 ± 2.6	12.5 ± 2.7
**Church attendance**	*n = 78*	*n = 14*	*n = 21*	*n = 23*	*n = 20*
≤1 time weekly	11 (14.1)	2 (14.3)	3 (14.3)	2 (8.7)	4 (20.0)
2–3 times weekly	14 (17.9)	1 (7.1)	4 (19.1)	5 (21.7)	4 (20.0)
4+ times weekly	53 (68.0)	11 (78.6)	14 (66.7)	16 (69.6)	12 (60.0)

*(n = ) in *italics* refers to valid n per question.

Data presented as n (%) for categorical data, and mean ± SD or median (IQR) for data that are normally and not-normally distributed, respectively.

#### Feasibility

Feasibility was assessed in relation to recruitment, scheduling, attendance and fidelity.

**Recruitment:** Members were mainly recruited through church announcements (rural setting) and word of mouth from the leaders (urban setting). A Pastor in the rural setting noted that women would typically ask a few questions before joining, but men needed a more personal approach from him. The urban church Pastors did not make announcements about the programme in church, partly due to the structure of their services and established practices for church-wide announcements; but this may also reflect a lack of leadership engagement in these churches. Programme leaders from Church 1 suggested that it can be difficult to involve people in new things without a particularly engaging hook, which they felt a lifestyle change programme does not have:

*[A]ny initiative that we have … always struggles in the beginning. It’s like we need a rock star present [laughter] to get people there. So that was my concern in the beginning… They were interested, but it may not have been like going to a concert…It’s like buying insurance. You know you need [it], but you’re not ecstatic about it*. *(Church 1 leader)*

To count as having ‘joined’ the programme, members had to have undertaken pre-programme measurements. By this criterion, 84 members were recruited, 68 of whom were recruited across the three churches that delivered the programme (Table 5). Most members were women (73%), unemployed (63%), frequent (≥ 4 times weekly) church attendees (68%), and classed as overweight or obese at baseline (74%) (Table 5). Mean BMI was 30 kg/m^2^.

**Scheduling:** The programme was abandoned in Church 1 following two attempts to run the first session with 16 members signed up, and on-going difficulties in securing a time that sufficient members could attend weekly sessions,. Church leaders were sorry but pragmatic, suggesting that a programme like this would be hard to implement,

… *one side says it could work*, *it’s relevant*, *it’s needed*, *there’s a place for it*. *But then I look at the fundamental issues…the challenges that we’re facing [with scheduling] and think that there’s going to be challenges somewhere in the implementation t… (Leader*, *Church 1)*

The other churches chose the most convenient day for the weekly programme sessions at their church. Even so, programme sessions often did not take place as scheduled. Several sessions were postponed, resulting in the 12 sessions being implemented over 16–20 weeks in Churches 2–4. Reasons mentioned in the focus groups and interviews for sessions not being held according to the planned schedule included: other church events or programmes taking priority; bad weather; funerals; month-end grant payment days; and problems with venue access.

…*there is no time where we can say it is right for all of us because we are different … especially as we are working*, *Saturday is the only day we are able to use… young are going to mall*, *you have to rush to the funeral*, *you are not able to attend*. *(Member*, *Church 4)*

Starting on time was also a problem in Churches 3 and 4. Members sometimes drifted into the session “*in drops*” well after the start, leading to problems in timing and incorporating key activities into the session. Fieldnotes from the rural setting highlighted members’ need to prioritise other activities or commitments, such as domestic chores:

*A few were on time*, *but other members were arriving in drops*, *as most of our members are females and have relatively more tasks to perform at home* … *The cultural division of labour [here] allocates all domestic chores to women; all these are factors that cause delays [*preventing*] our members [*from*] coming on time*. *" (Observation session 2*, *Church 4)*.

**Attendance:** In the churches in which the programme was implemented, only 43/68 members who were measured at baseline (63%) attended at least half of programme sessions. Many of the reasons discussed in relation to scheduling difficulties were also cited as challenges to regular attendance. Additional challenges included transport, illness, personal choice, and lack of motivation:

…*it’s a person’s choice at the end of the day*. *Sometimes you can’t force people to do what you want if they don’t want to… (Member*, *Church 2)*

Post-programme questionnaire data indicated the most common reasons for missing sessions included: too many commitments on that day, such as a funeral or work (42%); family or domestic commitments (28%), bad weather (16%), and transport problems (14%).

In the focus groups and interviews, leaders spoke about making efforts to encourage attendance, but that this did not always yield results, which could be dispiriting.

*They promise to come but they don’t*. *Even though you called a person and she confirmed the attendance*, *but she did not come*. *(Leader*, *Church 4)*

However, members noted, in particular in Church 4, that encouragement from the Pastor really helped with attendance.

**Fidelity:** We organise reporting around delivery of: physical activity sessions, information and delivery style, teaching behaviour change techniques, support for a mutually supportive atmosphere, using Christian practices of prayer and singing and whether Impilo neZenkolo Healthy Eating messages were delivered for members to share with family and congregation members.

The observation data suggest that group-based physical activity sessions were delivered with more consistency than ‘classroom’ sessions (in which the core components would be delivered). In Church 2, this is attributable in part to the fact that, from session 6 onwards, physical activity sessions were led by a research team member (ST), a qualified physical activity instructor who was present to observe the session but asked to lead it). In a later interview the Pastor at Church 2 suggested this was because the programme leaders lacked confidence in this aspect of the delivery, especially whilst being observed.

In Churches 3 and 4, group walking exercises were popular, and sometimes included song. Strengthening and flexibility exercises were well received in all three churches:

*After completing the walk*, *group members were asked what other activities they have started doing and how these are going*, *particularly the upper body exercises that they learned about the previous week*. *Most members commented that they find the exercises very helpful because they now feel lighter in weight*, *some even said they do really notice the change*. *(Observation*, *session 6*, *Church 3)*

The observation notes for this session also suggest that strength exercises were taught carefully by programme leaders,

*[the leader] made the group members try 10 repetitions for each movement*. *The strength exercises that were done included strength leg raise*, *assisted/supported stork stand*. *Members were told that ‘the most important thing is to ensure that they are doing the exercises correctly’*. *The rest of the group was counting the repetitions*, *so that they focus on their form*. *[The leader] spent 15 minutes on strength exercises*. *(Observation*, *session 6*, *Church 3)*

One common omission from in the group-based physical activity programme which consistently occurred across the three churches was the introduction of the rate of perceived exertion (RPE) scale in session 3.

The delivery of information content using interactive, adult-learning, techniques for sharing information was done well. Observations suggested that interaction was encouraged and lively interaction was observed, with some evidence of mutual and vicarious learning about: food preparation, fizzy drink consumption, using exercise to avoid stiffness, portion sizes, exercise intensity, and SMART goal setting. For example,

*[The leader] encouraged members to share their experience so that they support each other*. *There was so much laughter*, *members were very engaged (Observation*, *session 10*, *Church 3)*

Teaching behaviour change techniques varied. We found goal-setting featured in all three churches’ deliveries. In Church 2, leaders encouraged SMART goal setting for step counts, and consumption of food and fizzy drinks, although they tended towards a didactic delivery style, telling members what their goals should be, rather than encouraging them to develop their own goals. In the post-programme focus group discussion, leaders expressed their preference for didactic style teaching, perhaps because it was most familiar. Members at Churches 3 and 4 were encouraged to set self-identified goals for eating, drinking and step counts, although illiteracy inhibited recording goals at Church 3. SMART goal setting was particularly appreciated as a life-skill in Church 4:

*Setting smart goals helps a person to eat healthy and also makes a person to be organized*. *So people were happy for that and they started to think about taking smart goals*. *Also it allows those who learned to move on the next level and realize that they can take smart goals at work or anywhere*. *(Leader*, *Church 4)*

An omission at all three churches, however, was joined-up use of the SMART goal feedback loop; leaders frequently neglected to review goals from previous weeks and/or encourage members to set new goals for the coming week.

Self-monitoring appeared to be implemented with the lowest fidelity. Members should have been encouraged to monitor and record their weight and steps. Leaders in Churches 2 and 3 were seen to encourage use of the ‘personal progress record’ in conjunction with the pedometers but observation suggests that low literacy made it hard for some members to use the personal progress records as intended. Although members enjoyed being given pedometers, they experienced widespread problems with their use. In Churches 3 and 4 in particular there were many reports that pedometers did not work. In Church 2, use of scales for weekly self-monitoring of weight was only encouraged on 3/12 occasions, one of which was the ‘halfway down’ session, which included weighing as part of the mid-point feedback procedure. Churches 3 and 4 did not make consistent use of the scales because of persistent problems with the scales provided, a source of considerable dissatisfaction.

The fostering of a mutually supportive environment was observed in all churches. Members were encouraged to interact and contribute throughout the programme. Positive feedback was also apparent; members clapped and congratulated each other on their achievements, committed to supporting one another when experiencing setbacks and criticisms of their new lifestyles from others, and shared tips relating to cooking.

Prayers and songs were woven into the routine of the groups, as encouraged in delivery materials, and these familiar cultural forms provided members with opportunities to participate in and lead group interactions, and were much enjoyed.

*[The leader] asked one of the members to start a song; after singing another member was asked to pray*. *Members were asked to join in pairs and share with each other the scriptures and verses*. *They seem very much excited when doing this task*. *(Observation*, *session 3*, *Church 3)*

A disagreement between leaders at Church 4, possibly related to a larger rift in the church, hindered group interaction for a time, but a visit from church leaders provoked excitement in the group and seemed to improve group interaction. At Church 3, interactions between group members suffered when leaders seemed underprepared for the session.

Finally, it seems that the leaders emphasised passing on key messages. In focus groups members spoke about passing on key message to family and community members, e.g. neighbours, informal store owners; and integrating some of the programme’s messages into daily life in ways that affected others, such as purchasing healthier food for children or not giving children money for unhealthy snacks.

*So I have told them [my friends] like at least if you’ve got 20 Rands [±US$1*.*40] it’s a lot of money*, *you can buy like spinach*, *you can buy a lot of veggies and then you can make like a more healthy food out of that 20 Rand*, *and then you going to eat this food for lunch and you can keep it for supper as well or maybe if the kids are coming back from school they can eat from that food*. *Stop complaining that you don’t have money*, *it’s how you spend your money* … *(Member*, *Church 2)*

#### Acceptability

All church pastors acknowledged a need for the programme. Some specifically said it could address unhealthy eating and help to not “demonise” the acquisition of conditions such as hypertension and diabetes. None of the pastors had previous experience with health promotion programmes, although many had experience of other community outreach programmes through their church or community.

*we realise the need and the demand for the programme to be delivered was essential because there are lots of people who are sick due to unhealthy eating…there are many people as well who lose control of their bodies and they gained weight maybe due to unconsciousness of what they eating*. *(Pastor*, *Church 3)*

Programme leaders and members found programme materials helpful, and leaders were positive about the development workshops and training, seeing these as an opportunity to gain knowledge.

*The training was enough…I learn a lot about its expectations to be a trainer [leader]*, *to be a role model…I learn so many things and there are also many things that I believe that when times goes on I will learn more being part of it*. (Leader, Church 4)

The length of manuals was seen by some leaders to be intimidating for members, especially for those with literacy problems or unused to reading.

…*the book we use is very big*. *Sometimes you don’t know where to start*. *(Leader*, *Church 3)*

I did notice a couple of people violently paging through the book…It’s kind of thick…there’s a saying if you want to hide anything from a person put it inside a book. (Leader, Church 2)

Leaders and members expressed positive perceptions overall about the programme: how it was delivered, and how they had benefited from it.

*I loved it because it helped me in my life since there were things that I was failing to do while I am a Christian* …*I wasn’t aware about the impact of vegetables in our bodies*, *fruits and drinking water*. *I was drinking any kind of juice and drinks*. *I asked myself how I’ll afford this lifestyle since I’m unemployed*, *but they told me that I can use what I have but in a right way*. *(Leader*, *Church 3)*

…*you[‘re] the one who is responsible for your kids*. *If your kids get obese…you are the one who made them like that…because you’ve got the programme Impilo neZenkolo*, *so why your kids are so unhealthy*, *why are you still continuing to serving unhealthy food in your house but you are having a programme like that*? *So you understand it really helps us a lot*. *(Member*, *Church 2)*

In their responses to the post-programme questionnaire, participants (leaders and members) rated the programme highly (9.7/10, range 7–10). Members also rated programme leaders highly (9.4/10, range 7–10). Participants were positive about the usefulness of the programme (Table 6). The activity considered to be the most useful was exercising during the sessions (93%). Most (88%) who completed the post-programme questionnaire indicated they would be ‘very likely’ to continue being physically active after completing the programme.

**Table 6:** Participants’ views of aspects of the programme % saying ‘very useful’)*.

Aspects of the programme	n	Very useful
1. Using the pedometer for monitoring step count	43	38 (88.4)
2. Setting SMART goals for behavioural change	43	38 (88.4)
3. Filling in the Personal Weekly Progress Record	41	35 (85.4)
4. Understanding the cycle of life and how to overcome challenges	42	35 (83.3)
5. Bible messages specific for each week of the program	43	37 (86.1)
6. The programme being in church premises with other members of church	43	37 (86.1)
7. Using the walking program to increase physical activity	43	37 (86.1)
8. Comparing progress on the six-minute walk test	42	37 (88.1)
9. Becoming more active by making small changes to everyday life	43	38 (88.4)
10. Exercising with *Impilo neZenkolo* members during the sessions	43	40 (93.0)
11. Exercising with *Impilo neZenkolo* members in between the sessions	43	37 (86.1)
12. Exercising at home using the pictures and information from the *Impilo neZenkolo* Member Notes	42	37 (88.1)
13. Weighing on a regular basis	43	35 (81.4)
14. Using a food diary	43	37 (86.1)
15. Discussing the healthy eating plate to reduce portion sizes and choose healthier options within each category	42	37 (88.1)
16. Discussing drinking behaviour to limit intake of sugary drinks	43	37 (86.1)
17. Reading food labels to make healthier food choices	43	37 (86.1)
18. Planning to overcome setbacks	43	39 (90.7)
19. Having the observers attend some of the sessions	42	37 (88.1)

*Data presented as n (%) of participants that responded “very useful” to the statement

In the focus groups and interviews, the only topics that appeared to be less well received were alcohol consumption, and reducing red meat consumption. Leaders’ and members’ responses in the focus groups and interviews suggested that the opportunity to increase their knowledge was highly valued.

In addition to developing skills, leaders reported that their role as programme deliverers was manageable, although it required considerable commitment. For example, one said,

…*when we started delivering we were scared*, *but we learned*. *I learned to stand in front of people and to share information with others*. *So it helped very much*. *And ja [yes]*, *we enjoyed*.*(Leader*, *Church 4)*

Another key aspect of acceptability was the programme’s perceived alignment with the Christian faith; this was mentioned by all participant groups (leaders, members and church leaders). Many programme leaders and members articulated how bodily health fits into a holistic view of spirituality, and that this positively influenced their decision to participate. Programme leaders and members were also positive about the faith content and Christian ethos integrated into programme materials.

*It’s because they said*, *it’s ‘health through faith’ so that made us perceive that we will be assisted because it involves faith and there are also health related things…we were very much impressed*. *(Member*, *Church 3)*

…*that holistic version of spirituality goes hand in hand with having a healthy body…by this programme*, *this is one way that I can make God work through me*. *So it’s more spiritual to me to be involved in this programme than it is health conscious*. *(Leader*, *Church 2)*

… *it didn’t clash with that I am a believer*, *being involved on Health through Faith didn’t make me see the things that is against with being a brethren*, *so in other words it is going hand in hand… (Member*, *Church 4)*

It was evident from participants’ responses that the programme provided (or had potential to provide) a supportive environment within which members motivated each other. Participants spoke of a sense of belonging and enjoying being in a group whose members encouraged one another, with positive consequences for their motivation for lifestyle change.

*Working as a team … made it easier because you know that we meet on this time and we are many who are in this campaign of living the healthy life*, *that’s what encouraged us… it quite difficult to do a thing alone*, *it is easy when we are many*. *(Member*, *Church 4)*

#### Assessing potential effectiveness

Post-programme measurements were collected on 42/68 (62%) of participants from Churches 2, 3 and 4 that had baseline measurements. No-one from Church 1 (n = 16) was followed up.

In those with follow up measurements, there were significant improvements between baseline and post-programme in four objectively measured outcomes (weight, BMI, waist and hip circumferences) but not BP (Table 7; S1 Table for data by church). Weight loss averaged 1.3% of baseline weight.

**Table 7:** Participant pre- and post-programme objectively measured outcomes (participants with pre- and post-programme data)*.

	n	Pre	Post	p-value	
Systolic BP (mmHg)	40	123 (107, 132)	122 (116, 134)	0.085	z = -1.721
Diastolic BP (mmHg)	40	81 (72, 86)	84 (74, 92)	0.451	z = -0.753
					**95% CI**
Weight (kg)	41	80.5 ± 20.1	78.3 ± 19.1	0.010*	0.56–3.91
BMI (kg.m^-2^)	41	29.9 ± 7.4	29.1 ± 7.1	0.010*	0.20–1.42
Waist circumference (cm)	42	92.3 ± 17.4	88.2 ± 15.9	0.002*	1.53–6.51
Hip circumference (cm)	39	106.6 ± 13.9	103.7 ± 13.4	0.005*	0.94–4.92

*Data presented as mean ± SD or median (IQR) for normally and not-normally distributed data, respectively. P-values shown for differences between pre and post-programme measurements. Significant at p<0.05. z scores reported for non-parametric analyses; 95% confidence intervals reported for parametric analyses (difference between pre and post scores).

Significant changes included fewer participants reporting problems with mobility and usual activities post programme and an improvement in self-rated health; there was no change in measures of psychological distress or self-esteem (Table 8).

**Table 8:** Pre- and post-programme measures for self-reported health status, psychological distress and self-esteem (participants with pre- and post-programme data)*.

	n	Pre	Post	p-value	
**EQ5D health status**					**X**^**2**^ **(df)**
Mobility—n (%)	39				
*No problems*		33 (84.6)	35 (89.7)	0.043*	4.10 (1)
*Problems*		6 (15.4)	4 (10.3)		
Self-care—n (%)	39				
*No problems*		38 (97.4)	39 (100.0)	-	-
*Problems*		1 (2.6)	0		
Usual activities—n (%)	38				
*No problems*		35 (92.1)	36 (94.7)	0.023*	5.15 (1)
*Problems*		3 (7.9)	2 (5.3)		
Pain/discomfort—n (%)	39				
*No problems*		17 (43.6)	18 (46.2)	0.455	0.56 (1)
*Problems*		22 (56.4)	21 (53.9)		
Anxiety/depression—n (%)	38				
*No problems*		20 (52.6)	20 (52.6)	0.107	2.59 (1)
*Problems*		18 (47.4)	18 (47.4)		
					**z-score**
Current health	38				
*Score (out of 100)*		70 (50, 80)	80 (70, 98)	0.009*	z = -2.614
**K10 psychological distress scale**	40				
*Score (out of 50)*		16 (13, 20)	14 (11, 19)	0.198	z = 1.288
**Rosenberg self-esteem scale**	34				**95% CI**
*Score (out of 30)*		19.0 ± 4.5	20.1 ± 3.5	0.211	-2.98–0.68

*Data presented as n (%); and mean ± SD or median (IQR) for normally and not-normally distributed data, respectively. Chi^2^ analysis used for categorical data (for items with more than 1 valid group of variables), Wilcoxon sign-rank test used for not-normally distributed data, paired t-tests used for normally distributed data. P values shown for differences between pre and post-programme measures. Significant at p<0.05. Degrees of freedom reported for Chi^2^ analyses. Z scores reported for non-parametric analyses; 95% confidence intervals reported for parametric analyses (difference between pre and post scores).

The only improvements in dietary habits were decreased consumption of chicken with skin and chips/crisps, and increased fruit and vegetables score (Table 9).

**Table 9:** Pre- and post-programme measures for dietary habits (participants with pre- and post-programme data)*.

	n	Pre	Post	p-value
**Usually eaten foods–**n (%)				
Chicken/poultry	40			
*With skin*		17 (42.5)	7 (17.5)	0.003**
*Without*		22 (55.0)	33 (82.5)	
*None*		1 (2.5)	0	
Red meat	39			
*Fatty meat*		11 (28.2)	5 (12.2)	0.633
*Lean meat*		24 (61.5)	30 (76.9)	
*None*		4 (10.3)	4 (10.3)	
Spread	40			
*Butter*		7 (17.5)	4 (10.0)	0.059
*Hard margarine (brick)*		16 (40.0)	9 (22.5)	
*Soft margarine (tub)*		12 (30.0)	19 (47.5)	
*None*		5 (12.5)	8 (20.0)	
**Frequency of consumption–**n (%)				
Deep fried foods	41			
*Never*		8 (19.5)	6 (14.6)	0.296
*Occasionally/weekly*		33 (80.5)	33 (80.5)	
*Daily*		0	2 (4.9)	
Shallow fried foods	40			
*Never*		5 (12.5)	4 (10.0)	0.791
*Occasionally/weekly*		33 (82.5)	32 (80.0)	
*Daily*		2 (5.0)	4 (10.0)	
Chips (crisps)	40			
*Never*		8 (20.0)	7 (17.5)	0.043**
*Occasionally/weekly*		30 (75.0)	30 (75.0)	
*Daily*		2 (5.0)	3 (7.5)	
Processed meat	40			
*Never*		5 (12.5)	5 (12.5)	0.291
*Occasionally/weekly*		30 (75.0)	33 (82.5)	
*Daily*		5 (12.5)	2 (5.0)	
Breakfast	39			
*Never*		6 (15.4)	1 (2.6)	0.456
*1–2 times in the past 7 days*		11 (28.2)	8 (20.5)	
*3–5 times in the past 7 days*		7 (18.0)	13 (33.3)	
*>6 times in the past 7 days*		15 (38.5)	17 (43.6)	
**Fruit and veg score** (out of 70)	41	14 (11, 17)	18 (10, 28)	0.021**

*Data presented as n (%) where indicated; and median (IQR) for data that are not-normally distributed. Chi2 analysis used for categorical data, Wilcoxon sign-rank test used for not-normally distributed data. P values shown for differences between pre and post-programme measurements.

**Significant at p<0.05.

Pre- and post-programme results for self-reported physical activity and sedentary behaviour (measured by the GPAQ) are presented in Table 10. Both pre- and post-programme self-reported PA was very high, with 71% of participants reporting meeting guidelines at pre-programme, and 96% reporting meeting guidelines post-programme.

**Table 10:** Pre- and post-programme self-reported physical activity and sedentary behaviour (total sample)*.

	n	Pre	n	Post
**GPAQ–PA (min.wk**^**-1**^**)**	65		27	
Occupational MVPA		174 (0, 1485)		840 (180, 1380)
Transport MPA		60 (0, 420)		360 (0, 1260)
Recreational MVPA		0 (0, 240)		240 (120, 1200)
Total MVPA		570 (120, 2460)		1950 (900, 3975)
**GPAQ–SB (hr.d**^**-1**^**)**	61		27	
Sitting time		4.0 (2.0, 6.0)		2.0 (1.0, 4.0)

*Data presented as median (IQR) for all movement behaviour data (all not-normally distributed). PA = physical activity, MVPA = moderate- to vigorous-intensity physical activity, SB = sedentary behaviour

In the focus groups and interviews, participants reported a number of changes in physical health, some of which support the quantitative findings. Examples include: weight loss; improved fitness, mobility or ability to perform daily tasks; greater feeling of vitality; decrease in body pains; improved BP; being sick less often; fewer sugar cravings; and improved skin health.

Changes to behaviour were also discussed. These included: eating healthier food, decrease in portion sizes, increased fruit and vegetable consumption, not missing breakfast, drinking fewer sugar-sweetened beverages and more water, removing chicken skin, consuming less sugar, salt, starchy and fast food, using healthier cooking methods (e.g. less frying), increased physical activity (e.g. walking instead of driving, taking the stairs), walking faster, and managing stress better.

*I can say that Health through Faith helped us a lot…especially when I saw a lot of oil in food*. *It helped me a lot maybe to become active*, *I was walking not knowing how walking is helpful*, *just climbing stairs*, *and jogging*. *(Member*, *Church 4)*

I am staying away from all the, like the, junk food and all the takeaways and stuff like that…I just want to thank you guys for everything because I have changed a lot. (Member, Church 2)

#### Member recommendations for the *Impilo neZenkolo* programme going forward

Participants at all churches expressed a desire to have some kind of continuation of the programme. However, it was clear from their responses that this may need to be somewhat different in each church, and any future deliveries would require further adaptation drawing on lessons learned from implementation of the programme thus far.

In Churches 2, 3 and 4, issues around resources featured in all their recommendations for future plans for the programme. In the rural setting, the continuation of the programme seemed to be linked to a desire for the research centre to keep providing funding the programme and the need for resources for delivery. These included remuneration for leaders, and incentives for participation for members such as transport costs and refreshments during the sessions. At Church 4, a request was made for ongoing training and mentorship during delivery.

At Church 2, programme members acknowledged that this type of programme usually comes at a cost to members, so they appreciated getting it at no cost. Leaders suggested a small fee could be charged for the programme so that it is would be perceived to have value, and ensure attendance. Leaders at this church also recommended that the programme is delivered in a more didactic style, and that members have “homework” to keep them accountable.

To help recruitment participants suggested advertising the programme more widely in and outside of the church, having pastors actively promote the programme and having an information session for interested people prior to recruitment. For example,

*Ideally*, *I would have loved us to have had a*, *a launch of some sort…Something interesting*, *something that can grab people’s attention from the beginning*, *and*, *and inspire them to start…something that is*, *um*, *catchy to the eye and engaging their interest…I’m all for big impact*, *for something that is ‘showy’*. *I sense people need that to move*. *(Leader*, *Church 1)*

Some participants felt it would be easier to promote the programme now that there was a clearer idea of what it involves. Leaders in the rural areas also supported the idea of leaders being paid a stipend which they felt would help professionalise the programme and ensure attention to detail in delivery.

## Discussion

This paper has reported on the development and initial evaluation of *Impilo neZenkolo*, a healthy lifestyle programme for low-income, black South Africans, delivered through churches. Aspects of the programme appeared highly acceptable in these settings, particularly the way in which the health focus of the programme was aligned with principles consistent with a Christian ethos. There was also some indication that, amongst those we were able to follow up, the programme can potentially support weight loss. However, other findings suggest that it was not feasible to deliver the programme as designed, i.e. over 12 weeks by unpaid church community leaders with limited time for training.

The main challenges to feasibility were: recruitment of churches in the urban area; scheduling of programme sessions; programme attendance; and some aspects of programme fidelity. Research has shown that community readiness to initiate church-based health promotion programmes is low in SA.[14]

In relation to recruitment of churches, following guidance developed in the USA,[22] we identified churches locally. It is possible working with national leadership in common Christian denominations may have helped, as would greater, more professional, marketing of the programme and resources.

Despite recruiting enthusiastic leaders and 16 members interested in the programme, scheduling of sessions was a problem in one church in the urban setting which ultimately did not deliver InZ. It may be that with other responsibilities (including paid work, family commitments, other community activities), congregations in urban areas do not yet have an appetite for healthy lifestyle programmes. In the rural setting, it was not difficult to recruit members, perhaps because of high unemployment, although other commitments again caused problems with attendance and retention (see below). Previous research in SA has shown rural settings can be very receptive to community-based interventions despite considerable structural barriers associated with low levels of socio-economic resources.[45]

Programme attendance was a problem. Programme membership was hugely fluid, and in rural churches members would often arrive late, leading to problems with accommodating the full session content, possibly reflecting accepted practices for other church activities such as Bible study groups. Given the target population’s socioeconomic circumstances, reimbursement for travel and the provision of refreshments, refreshments may improve attendance and this was encouraged by church members in focus group discussion. Emphasising the importance of regular and on-time attendance might also help but transport challenges may militate against this. It is also possible that a different sort of programme which allows ‘dropping in, and more individualised one-to-one support may increase attendance, but would be more resource intensive and would lose the valued benefits of group-interaction and mutual support which we knew to be important in our football based programmes.[27]

Although programme leaders reported liking the idea of SMART goal setting, fidelity was a problem for this and other behaviour change techniques despite additional training specifically on behaviour change in the rural setting. Teaching behaviour change is a skilled activity and we had planned two full days training for to cover this. However, in the urban area leaders insisted on a maximum of two, two-hour, training sessions because of their other commitments. We think this may have contributed to lack of detailed understanding of the programme and problems with fidelity. A recent systematic review of obesity interventions in African American faith-based organisations found some evidence that programmes led by lay health advisors were less effective than those led by research organisation staff. [19] Authors suggest that as in the present study some reasons for this might be a need for better training, and, potentially the need for adequate compensation for time. Another contributory factor in this study may include low levels of literacy in rural areas and the need to limit the programme’s resource costs because of our grant funding.

Urban church leaders expressed desire for a more didactic style of delivery reflecting familiar styles in the South African setting, where learning environments can remain outcomes-focussed and student-centred learning difficult to implement. It is possible that better and longer training could challenge these views, although as we have seen, a volunteer unpaid model is unlikely to support longer training.

Despite implementation challenges the programme proved acceptable and showed some potential for effectiveness. Outcomes with the most potential for change include those relating to body weight and size, perceived health status, mobility, daily tasks, and certain dietary habits. Although it was a significant component of the programme, physical activity was harder to measure and monitor and the use of affordable, but potentially less reliable, pedometers presented challenges as an intervention tool in these settings. While the group-based physical activity sessions were observed to be enjoyable, the programme’s nutrition component seems to have resonated more with participants, based on the focus group and interview data.

The overall aim of our research was to consider whether churches, as existing culturally valued settings, served as an adequate ‘hook’ for attracting people to a health-promoting group-based lifestyle programme. As with research on church-based health promotion in the USA,[19–22] we have demonstrated potential for South African church congregations to embrace health promoting programmes and achieve positive outcomes. We have shown that the ‘hook’ worked and many features of the programme were acceptable, appropriate and feasible to deliver. However, we have also shown that the programme was not straightforward to deliver and recommend further modification prior to further evaluation (see Table 11 for a summary).

**Table 11:** Suggested modifications to *Impilo neZenkolo* prior to further evaluation.

Problem encountered	Suggested modification to a revised programme
Recruitment of local churches	Programme developers work with national church leadership to endorse and promote programme.
	Programme developers work to identify potential programme funders at national or regional levels to support more professional, marketing of the programme.
Recruitment to, and attendance at, the programme	Offer contribution to travel and refreshments at sessions.
	Emphasise benefits of regular and on-time attendance to achieving outcomes Consider and test a ‘drop-in’ model of delivery.
Poor fidelity	Consider the development of professionalised, paid, network of leaders rather than unpaid volunteer leaders from the local congregation. Recruitment could specify experience in leading physical activity and congruence with faith-based ethos.
	Paid leadership may enable provision of more detailed, longer training. Quality assurance and on-going support for leaders to better support fidelity. Ensure sufficient funds to purchase reliable materials for self-monitoring (pedometers, scales) and support church costs.

Our study has notable strengths. It remains one of the first to develop and fully describe the delivery of a community-based healthy lifestyle programme for low-income black South Africans. Our development approach helped ensure the programme’s acceptability despite the implementation problems described. We used mixed methods to consider the feasibility, acceptability and potential effectiveness of the programme, allowing triangulation of analyses and confidence in our conclusions.

We also note some limitations. The study relied on a convenience sample of churches identified through the personal networks of one research team member in the urban setting. As a feasibility study, it was not possible or appropriate to randomise either churches or participants and we did not have a comparison group. This does, of course, limit any generalisability of potential effectiveness. We also encountered problems with retention in the study which suggests that subsequent research will need to be well enough resourced for robust follow-up. Although the Global Physical Activity Questionnaire (GPAQ) is commonly used in SA,[37] is recommended by the World Health Organisation for surveillance of physical activity levels and has been reported as suitable for measuring changes in activity,[46] we did not have confidence in the data it produced in our study. There are two possible explanations: it is possible that we paid insufficient attention to training in administration of the GPAQ; and/or despite training and explanations provided during GPAQ administration, participants reported high levels of physical activity, which may be real or because of problems with interpretation. In addition, it is possible that some of the self-reported outcomes may have been subject to social desirability bias.

In conclusion, despite substantial challenges to implementation, we think a healthy lifestyle programme for low-income, black South Africans, delivered through churches, may be viable with extensive re-development of delivery strategies to address the challenges we encountered.

## Supporting information

S1 Table Participants’ pre- and post-programme results for objectively measured outcomes, for total sample and by church. (DOCX)

S1 File Further qualitative data extracts. (DOCX)
