# Supplementary material for: Development, feasibility, acceptability and potential effectiveness of a healthy lifestyle programme delivered in churches in urban and rural South Africa
Source: PLoS One. 2019 Jul 31;14(7):e0219787. doi: 10.1371/journal.pone.0219787 (PMC6668772; doi:10.1371/journal.pone.0219787)
Supplement: S1 Table — (DOCX) [file pone.0219787.s001.docx]

**S1 Table.** Participants’ pre- and post-programme results for objectively measured outcomes, for total sample and by church*

|  | **Total** | | **Church 1+** | **Church 2** | | **Church 3** | | **Church 4** | |
| --- | --- | --- | --- | --- | --- | --- | --- | --- | --- |
|  | **Pre (n=83)** | **Post (n=42)** | **Pre (n=16)*** | **Pre (n=23)** | **Post (n=11)** | **Pre (n=24)** | **Post (n=17)** | **Pre (n=20)** | **Post (n=14)** |
| **Systolic Blood Pressure** | *n=79* | *n=42* | *n=16* | *n=19* | *n=11* | *n=24* | *n=17* | *n=20* | *n=14* |
| (mmHg) | 118  (103, 127) | 122  (116, 134) | 100  (90, 108) | 122  (107, 132) | 121  (117, 137) | 118  (106, 129) | 116  (101, 123) | 121  (111, 128) | 128  (126, 138) |
| **Diastolic Blood Pressure** | *n=79* | *n=42* | *n=16* | *n=19* | *n=11* | *n=24* | *n=17* | *n=20* | *n=14* |
| (mmHg) | 77 (69, 85) | 84 (74, 92) | 65 (61, 72) | 77 (69, 92) | 86 (76, 91) | 79 (72, 85) | 74 (62, 85) | 85 (76, 91) | 88 (84, 94) |
| **Weight** | *n=83* | *n=41* | *n=16* | *n=23* | *n=11* | *n=24* | *n=17* | *n=20* | *n=13* |
| (kg) | 79.3 ± 18.3 | 78.3 ± 19.1 | 74.2 ± 14.9 | 81.9 ± 21.6 | 79.9 ± 21.9 | 74.5 ± 17.0 | 73.7 ± 16.9 | 86.0 ± 17.0 | 83.0 ± 19.5 |
| **BMI** | *n=81* | *n=41* | *n=16* | *n=23* | *n=11* | *n=22* | *n=17* | *n=20* | *n=13* |
| (kg.m^-2^) | 30.1 ± 7.0 | 29.1 ± 7.1 | 29.3 ± 6.0 | 30.4 ± 8.3 | 29.6 ± 8.3 | 29.9 ± 7.3 | 28.4 ± 6.7 | 30.7 ± 6.2 | 29.6 ± 7.2 |
| **Waist circumference** | *n=83* | *n=42* | *n=16* | *n=23* | *n=11* | *n=24* | *n=17* | *n=20* | *n=14* |
| (cm) | 91.2 ± 16.6 | 88.2 ± 15.9 | 85.0 ± 16.4 | 91.1 ± 19.5 | 86.5 ± 16.6 | 91.5 ± 13.6 | 90.0 ± 15.0 | 96.0 ± 15.9 | 87.4 ± 17.3 |
| **Hip circumference** | *n=80* | *n=39* | *n=16* | *n=22* | *n=10* | *n=23* | *n=16* | *n=19* | *n=13* |
| (cm) | 106.6 ± 14.0 | 103.7 ± 13.4 | 102.3 ± 11.1 | 109.7 ± 15.9 | 108.0 ± 14.2 | 106.0 ± 14.5 | 103.2 ± 11.9 | 107.6 ± 13.1 | 100.9 ± 14.7 |
| **(n=)* in *italics* refers to valid n per measurement. Data presented as mean ± SD or median (IQR) for data that are normally and not-normally distributed, respectively. + Church 1 has no post-programme data for any measurements as the programme was not delivered in Church 1. | | | | | | | | | |
