# Supplementary material for: Development, feasibility, acceptability and potential effectiveness of a healthy lifestyle programme delivered in churches in urban and rural South Africa
Source: PLoS One. 2019 Jul 31;14(7):e0219787. doi: 10.1371/journal.pone.0219787 (PMC6668772; doi:10.1371/journal.pone.0219787)
Supplement: S1 File — (DOCX) [file pone.0219787.s002.docx]

# S1 File. Further qualitative data extracts

## Feasibility

### Recruitment and scheduling

| Members were mainly recruited through church announcements (rural setting) and word of mouth from the leaders (urban setting). | |
| --- | --- |
| FGD Church 4 (rural) | *Note, the Interviewer (I) respectfully referred to participants as ‘mother’, ‘father’, ‘aunty’ and so on.*    I: Okay, the first question [I want to ask is]… how did you hear about the program? Did you have enough information about it before you start?  P2: I heard here at church that there is a program like [InZ]… and I think the information we got was enough that… it was enough that we can be a part of this program [to make us want to join].  I: What do others say…? Yes mother.   P4: I heard pastor introducing this program, he addressed it here at the church, and he passed this information to the congregation saying… where he got this information from [the research team]  I: Okay, you can speak my father.  P5: Yes… yes, I heard about this program here in the church as they have said, I also heard about it clearly when the research team joined our service. I understand it [what it was about] and decided to join.  I: Okay, mother you can talk…  P3: Ooh… I was also going to say that I heard about it here at the church by the pastor, when he began explaining about it I understood it, I also heard from his explaination that they need volunteeres who will be trained to manage this program, so I heard about it here at church and I loved it. I: Eerh is there anyone who wish to add or you all have same answers.  P(ALL: They are same… |
| FGD Church 2 (Urban) | P1 I just heard about the programme through one of our volunteers, [name of leader]…he is the one who came up to us after the [church] meeting and said “there are people who are just running a programme”. I was not understanding I was “what?”, “what is that?”. ……I want to pray about it at home after church [and I did, so] really then when I come the next Sunday, I just wanted to try it, you know actually it all was in my heart.  P2 Okay for me … It was a member of the church, [who told me]. He just told me “there is a programme that …that you may find that it’s very interesting, it’s about Impilo neZenkolo, it’s about your…your…like…uuh…they actually tell you how to deal with yourself about physically and uuh…spiritually, mentally and all things [health]. It’s all one actually. So I said okay fine I would love to know, especially when it’s uuh…physical it’s like you are going to exercise. |
| It can be difficult to get people interested in new things (in urban area) | |
| Interview with Leaders Church 1 (Urban) | I: Ja. Did you feel it was difficult to get people recruited, so people, you’d tell them about it and they say “nah, I’m not interested”   Ldr : I, I think they did [find it interesting]. They were interested, but it may not have been like going to a concert, type of thing... [laughter]. You know what I mean? It’s like buying insurance. You know you need, but you’re not ecstatic about it. [I: Ja] So, it’s that kind of interest. You know this is good for you, and you recognize it is something that you should be doing it, but it does not light up anything. Ja, that, that’s what I think. |

### Scheduling

| Scheduling | | |
| --- | --- | --- |
| Interview with Leaders Church 1 (Urban) | I: (asking why scheduling was such a problem. … in some churches we have heard that funerals take up a lot of the free time on the weekend and…  Ldr 1: And remember when it comes to funerals, it’s not just the Sunday that is taken, .., it’s the week or two leading up to that Sunday. So, when you come [back from] work, there are daily prayers that you attend at that home. [That means] , so whatever it is that you [usually so] during the week to do, um, you put off for the weekend. But then you get to the weekend, and you can’t use the Sunday [to do chores] because you have to go to the funeral. [I: yes]]. So, you have to force it on that weekend to make it happen. ….… more than five weekends this year alone, that we’ve had to go to funerals. | |
| FGD Church 4 (Rural) | I: Ok, the next question is, tell me how the church decided to deliver the program?  P1: …they chose to use Saturdays, they realized that it distracting during the week.  I: Ehhhh  P1: They chose weekends…even the weekends give a problem because we have funerals but we try to make it afternoon….we trying to cover everyone. | |
| Starting on time could be a problem in Churches 3 and 4 | | |
| Field notes from observation of week 7, Church 4 | | The program was meant to start at 13:00 but members don’t always come in time for the program delivery. Two members came in very early and waited in their car, two other members came in at about 13:28. While we waited to hope that members will come in numbers, [a leader] was also having a conversation with other members about the InZ program. Members came in drops, they were seated in a circle set up. |
| Field notes from observation of week 7, Church 3 | | There were 15 group members on this day of the delivery, amongst the group there were also four people who are above the age criteria but still attend the program and 18 absentees. Members were coming in drops …. They seem happy to see each other again on the 6th week of the program delivery." |
| Attendance was sometimes a problem with low numbers attending | | |
| Interview with leaders Church 1 (Urban) | I: Ja. And were transport challenges a problem?   Ldr 1: Yes.  I: I mean, people saying “we can’t get there” or “I don’t want to commit because I don’t know how to get there”?  Ldr 1: It’s that, it’s just that our members are quite scattered.   I: OK  Ldr 1: So, they not all come from this part of, of [the urban area]. They’re coming from all over. So, we, sometimes they maybe think about even the journey to go, may not be... it might be putting them off. You understand? And um, maybe as a result of that they might, I think it contributed [to lack of attendance].  Ldr 2: It’s just we do not have other place where we can meet. That is the only place that we can meet. So, meaning that they do not want to compromise. | |
| Interview with leaders Church 3 (Rural) | I: What were the barriers you faced to deliver InZ?  P1: eh the hardship we faced was to see that there were no attendance, eh you find out they come sometimes they do not come due to certain situations.  I: mhmm  P1: eh due to certain situations like …. people were getting married, there are funerals eh all that, but some, they kept on coming.  I: ok  P1: but I would say it was the main challenge we had.  I: mhmm maybe that members were coming late?  P1: people coming late mhmm that was it [a problem].  I: mhm what…were there any practical problems during program delivery?  P1: mhm the practical problems we had was…  I: ehee  P1: … the poor attendance and late comers but we managed to sort that one by allowing them to contact me personally.  I: ok  P1: We have seen the difference…  …… I: mhmm ok eh were there anything, events were in the church or in the environment that may influence how the program worked in the church? As you explain father that people were attending…can you please list things that stops people from attending the program?  P1: mhmm eh others were staying far from here (inaudible)…if there is no car coming this side to give him a lift, he does not come to attend. | |

## Fidelity

### Physical activity sessions were usually delivered well

| Field notes from observation of week 6, Church 2 (Urban) | The lower body strengthening was led by the researcher and done well of course. The members seem to enjoy the exercises, and were motivating each other to keep going. During the squats, [name of member] said to the group: “I need this.” [name of leader] kept count during the stork stand exercises. |
| --- | --- |
| Field notes from observation of week 9, Church 4 (Rural) | There was some laughing and joking in this session. When asked about whether they can do the strength exercises at home, Many laughed, they started talking with one another I overheard one member saying the only that works for walking at moderate intensity when time allows her to do so. Group members were asked if they felt that the exercise session more difficult. Many were complaining that the exercise was difficult but they think it will help them in the long run. |
| Field notes from observation of week 6, Church 3 (Rural) | The PA seems to be going down well. After completing the 6-minute walk, group members were asked what other activities they have started doing and how these are going, particularly the upper body exercises that they learned about the previous week. Most members commented that they find the exercises very helpful because they now feel light in weight, some even said they do really notice the change which is absolutely a good thing.  Doing strength exercises they counted with each other. They performed 10 repetitions for each movement. The strength exercises that were done included (strength leg raise, assisted/supported stork stand). Members were told that for this initial session, the most important thing is to ensure that they are doing the exercises correctly. The rest of the group was counting the repetitions, so that they focus on their form. The leader spent 15 minutes on strength exercises. |

### Information and delivery style

| Field notes from observation of week 8, Church 2 (Urban) | There was good interaction in discussion about social influencers and later, when one said how difficult it was to get her toddler to drink water, all group members understood and were supportive . The lady said, "*At home I have a toddler and an older child. I’m always the person saying “drink water. I’m boring. To tell them what to do and change their mind set is not easy*." The group agreed with this challenge, with the leader saying “*You’re going to be a toothache when you’re an influence. Or a tongue pain.*” Members agreed with his analogy. |
| --- | --- |
| Field notes from observation of week 3, Church 4 (Rural) | Interaction was well supported. When they came in the members shook hands and were happy to see one another. They also discussed progress with healthy eating goals and leaders asked for suggestions as to how they would increase the intensity of their PA, members mentioned that from now on will try and increase by walking at a moderate level when doing their chores in their houses. |

### Teaching behaviour change techniques

| Field notes from observation of week 3, Church 2 (Urban) | Setting SMART goals was done, but leaders were pretty didactic – information giving rather than elicitation of goals. Leaders told members “Every Sunday you are to come in and weight yourselves”. [name of leader] said, “You alone are responsible for your success”. [name of other leader] recapped the weekly plan for increasing step count. Members were rold to make sure that they are increasing their steps every week. |
| --- | --- |
| Field notes from observation of week 3, Church 4 (Rural) | Review of SMART goals was done well. The eating goals were reviewed as planned including what had gone less well. Members mentioned that they sometimes find it hard to balance InZ lessons and having to take care of their families but also promised that since the InZ messages will help lead a healthy lifestyle they will keep trying" Also, they set new goals, although may have been didactic [name of leader] further encouraged group members to set SMART Goals as it will help them to be more focused and determined to balance eat-well and physical activity. Members were also told to engage in sport. |

### Mutually supportive environment

| Field notes from observation of week 4, Church 4 (Rural) | Since there were three members attending the session, they took turns in sharing with the rest of the group what worked well. [This] included the ability to set achievable goals and trying to practice the lessons learned from the InZ sessions. I [the fieldworker] was also invited to share my testimony which included setting achievable eating and physical activity goals, I also included the specific plan for both (eating and physical activity goal) group members seem engaged and inspired. |
| --- | --- |
| Field notes from observation of week 2, Church 3 (Rural) | They did the ice-breaker activity this week, first, which they enjoyed. The leader asked one of the one member to start a song, after singing another member had to pray. Members were asked to join in pairs and share with each other the scriptures and verses. They seem very much excited when doing this task because they were involved. |
| Field notes from observation of week 10, Church 2 (Urban) | There was good interaction about problems members are encountering in eating healthily and some shared experiences of solutions |

### Integration of faith through prayer and song

| Field notes from observation of week 4, Church 4 (Rural) | At the beginning, after praying [the leader] asked members to stand up and she began a song which made everyone dance and all three members were happy with the singing and doing a bit of physical activity which also made them to sweat |
| --- | --- |
| Field notes from observation of week 5, Church 2 (Urban) | No prayer at beginning today because they'd just come from church and been blessed already. But faith really brought into the discussion today in relation to food. [name of leader] started the discussion about the biblical perspective on food labelling (on page 38 in the manual). Quotes [name of leader] used during this included: “There is temptation, even with food” and “I can’t eat everything that people show me”. He went on to say that God provides us with many ingredients in natural foods, and that we should not buy things that have these extra ingredients. |
| Field notes from observation of week 6, Church 3 (Rural) | Prayer seen as one way to overcome setbacks. For example, in discussing how they overcome setbacks during the 'cycles of life' discussion, the leader said, the only setback is the negativity they keep getting from the people who are not part of the InZ. The only way they have dealt with it was through prayer and sharing words of wisdom with each other. |

### Passing on key InZ messages

| Field notes from observation of week 7, Church 4 (Rural) | Group members were asked to tell at least one other person one key message: use less oil during cooking to improve the health of your family. Other members even confirmed to the rest of the group that they now practice this important InZ message as well as other messages which made me feel happy because it showed that they understand and take everything we do very important. |
| --- | --- |

## Acceptability

| FGD Church 4 (Rural) | P5: Yes, when I heard about this program, I saw that I would help me as a believer. I saw that it involves faith/religion and saw that if it explains about healthy diet, physical exercises, I knew that it would help me. Although I was exercising, I was not eating [a balanced diet] in a way they have explained here. [We learnt} that …if [you] eat according to the way they explained you become healthy.  I: Is there anything anyone would like to add?  P8: Ay… I hear her… I just like it (the InZ programme]. It helps a lot. It also helped me personally as an individual, I am happy that congregation also like it and I can see that it helped them. |
| --- | --- |
| FGD Church 3 (Rural) | P5: It’s because they said, it’s health through faith so that made us perceive that we will be assisted [it wioll help] because it involves faith, and there are also health related things, we were taught about the way we eat, and we were very much impressed. |
| FGD Church 3 (Rural) | P2: …we liked everything, there is nothing [we did not like]…in my point of view everything was right…we liked it, we were happy that we were exercising  P7: Sometimes even if you ask us to stand with one foot as we are old now, but we are doing it, we can be able to push…it is happiness we do it joyfully.  P2: We are happy, even if you see that you are left behind [you are last in a walking group], you will run and you will laugh when we reach them. We will laugh and we are happy and free.  P(ALL): It’s interesting, you shouldn’t stop us from playing the ball [you should include us playing ball games]…we even like playing soccer. |
| FGD Church 2 (Urban) | So in the end I ended up falling in love with what was happening each and every week, I don’t want to lie to anyone. I was the one who was against this, but at the end of the day also it helped me and I mean there’s a lot of changes that I have experienced now in my life and everything. Also and I would actually commend it to most people from the church, and I would also be willing to go also all the way even to help other people also, who are running also against the idea negative. I am so much positive I don’t want to lie. So I am so happy. |
| FGD Church 2 (Urban) | So I find it like maybe it’s uuh…I benefited a lot. So ja, that’s how I can say, I found it… it was a really a very good programme for me, so it was very interesting. |

## Reports on effectiveness

| FGD Church 2 (Urban) | I was seeing the difference [each week]. Because of first of all I didn’t know how to eat healthy food, I was used to eating junk food most of the time and I was telling myself that to buy fruit and vegetables is expensive. And immediately I hear here in the group that, no those things are not expensive, I have to buy it if I want to change the way I eat. |
| --- | --- |
| FGD Church 4 (Rural) | P1: Eerh I can say the program [health through faith] helped a lot, because I had belly fat… . Now, when I am at work I walk a lot - that makes me stay health. Now we even reduce the amount of food… I am now used to limiting the [amount] food. They have agreed at home that they must only two dish spoons maybe of food and [I have been] eating vegs. My kilograms have decreased from a high number, I am just [feeling] healthy. I feel young and fresh. |
| FGD Church 3 (Rural) | P2: I think there is a difference, I liked food, I was eating too much. But, due to health through faith… I’m now eating half, you see, I’m now no longer eating 4-5 slices of bread. I’m now eating two slices of bread, I drink one cup and go to do my activities, if I feel that I’m hungry I take one slice again. They didn’t say I must eat too much eh…they advised me to take small amount of food at a time so that everything will be okay, I can feel the difference, I’m not getting hungry now and I’m happy for that. |

## Member recommendations for Impilo neZenkolo going forward

| Content of the programme | |
| --- | --- |
| Interview with leaders Church 2 (Urban) | Ldr 1: So that’s one thing but I think the point of departure should be the conditions that people [can get with lifestyle, we should use]... the fear factor there.  I: Okay so the health conditions? Ldr 1: Mmm.  I: Okay.  Ldr 1: Ja, so that you say when you are a diabetic what kind of lifestyle you live when you are on this and then you sort of reconcile all those that they all take us back to this programme you see. That’s the impression we want to give them, that this programme actually balances all those together as one solution, you see? |
| FGD Church 3 (Rural) | P5: To add on what others have said, I would suggest that maybe if we can have something that we can see with our eyes as we are learning about food. Like fruits, so that we will know that we should eat fruits after each and every meal. It would be better if one day we should plant fruit trees so that the young ones will see as they grow up and say ‘our mothers, when they were in church they were taught to eat fruits, this is their tree which they’ve planted’. Another thing is that if we can get donation for these trees so that we can plant them in our households and that will be good, even if our neighbours ask as to how did we find this tree, I will tell him/her that I got it through joining health through faith at [the church] so that we can have things to point at. |
| Interview with leaders Church 4 (Rural) | I think as we are saying that, for example, eh a person can see you using some of the things you are teaching them about. [For example, if you brought apples they could], also learn that you don’t need many apples but can have shares in everything. If you brought apples let us cut it into many pieces so that everybody will get equal share. |
| Delivery | |
| Interview with leaders Church 2 (Urban) | Ldr 1: Which takes us back to that drilling [learning] part again, you see, it needs to be done as homework.  Ldr 2: Ja, exactly, because when you tell them that “oh guys we’re going to meet next week, please don’t forget to fill in the books because there will be a day that we’re going to need to check the books”.  I: Okay and, you mean have a test?  Ldr 2: Ja, when we want... so you give the chance to visit [help] one another, “hey do you know what’s going on?, No here it’s like this and this, Me I’ve got a problem of this page, no this one knows this page”. So they’re trying to combine their heads [understaning] before they meet us and [by doing their homework] you see?  I: Okay. …..So almost running it more as like a course than a programme that you kind of the leaders....  Ldr 1: Yes, yes. ….You got me now!  I: Okay but you’re not going to scare people away?  Ldr 2: No.  Ldr 1: No, they... they want to see, just to be seen to be more educated than the rest.  I: Okay, so more educational? |
| FGD Church 3 (Rural) | P2: What I am saying eerh…to our leaders… they should fetch those people who has problems… the work should be continued…and we should bring people here |
| Interview with leaders Church 4 (Rural) | Yes, I think the things that I feel it was left out. That is , we didn’t have games in Health through Faith. We didn’t get to challenge each other maybe like through playing netball, playing drama eh just football, soccer ball things like that eh? or maybe the run fun walks together.  I: Oh  Perhaps in the future one day we can add the community [and ask] if they would like to join us. We could have a fun run walk from here to there, maybe, like turning back from town and come back here. |
| Incentives | |
| FGD Church 3 (Rural) | P2: My child I don’t want to tell lies, after I heard about the program, I didn’t expect money, I expected nothing. But after we have started exercising I then said, “if they can provide us with clothes that we can wear when exercising”, because we can’t exercise wearing nothing. It will be good if we can have our own hall and be provided with track suits and exercise inside the hall you see, if they can build a hall for us it’s where they are going to see old ladies exercising, walking here in the yard and the community will see us, even the one who didn’t like it now will join this thing. |
